# Protective effect of *Luffa cylindrica* Roemer against dexamethasone-induced muscle atrophy in primary rat skeletal muscle cells

**DOI:** 10.1007/s10974-023-09661-5

**Published:** 2023-10-17

**Authors:** Changhwan Yeo, Hyunseong Kim, Wan-Jin Jeon, Junseon Lee, Jin Young Hong, Hyun Kim, Yoon Jae Lee, Seung Ho Baek, In-Hyuk Ha

**Affiliations:** 1 Jaseng Spine and Joint Research Institute, Jaseng Medical Foundation, Seoul, 135-896 Republic of Korea; 2 College of Korean Medicine, Dongguk University, 32, Dongguk-ro, Ilsandong-gu, Goyang-si, 10326 Gyeonggi-do Republic of Korea

**Keywords:** Glucocorticoids, Muscle atrophy, Dexamethasone, *Luffa cylindrica* Roemer

## Abstract

Glucocorticoids (GCs) are commonly used in the treatment of chronic inflammatory conditions. However, the administration of high doses and long-term use of GCs can induce muscle atrophy (MA) in patients, leading to a decline in quality of life and increased mortality. MA leads to protein degradation in skeletal muscle, resulting in a reduction of muscle mass. This process is triggered by GCs like dexamethasone (DEX), which induce the expression of E3 ubiquitin ligases, namely Atrogin-1 and muscle RING-finger protein-1 (MuRF1). In this study, we examined the anti-MA potential of *Luffa cylindrica* Roemer (LCR) on DEX-treated primary skeletal myotubes. Primary skeletal myotubes stimulated with LCR alone resulted in a significant upregulation of myotube development, characterized by an increase in both the number and diameter of myotubes. Contrastingly, combined treatment with LCR and DEX reduced the expression of Atrogin-1, while treatment with DEX alone induced the expression of MuRF1. Furthermore, LCR treatment successfully restored the number and diameter of myotubes that had been diminished by DEX treatment. These findings suggest that LCR holds potential for treating MA, as an accelerating effect on muscle development and anti-MA effects on primary skeletal muscle cells were observed.

**Supplementary Information:**

The online version contains supplementary material available at 10.1007/s10974-023-09661-5.

## Introduction

Muscle atrophy (MA) refers to changes that occur in muscles as a result of various pathophysiological stimuli, including aging, starvation, muscle inactivity, and diseases. It is characterized by a reduction in protein content, fiber diameter, and force production (Jackman and Kandarian 2004). MA not only leads to decreased functional capacity and weakness in patients but also contributes to obesity, thereby negatively impacting the quality of life and increasing mortality rates among affected individuals (Foletta et al. 2011; Marcell 2003).

Glucocorticoids (GCs) are a class of steroid hormones known for their potent anti-inflammatory and immunosuppressive effects. They are commonly prescribed for the treatment of chronic inflammatory diseases like systemic lupus erythematosus, rheumatoid arthritis, and bronchial asthma (Troncoso et al. 2014; Ma et al. 2003). However, the prolonged and high dose use of GCs can result in MA and frailty due to their catabolic effects on skeletal muscle tissue (Hasselgren 1999; Hermans and Van den Berghe 2015). In muscle cells, excessive GCs bind to the GC receptor, triggering MA. This process involves the upregulation of specific E3 ubiquitin ligases, such as Atrogin-1/muscle atrophy F-box and muscle RING-finger protein-1 (MuRF1), which play a role in GC-induced MA (Mishra et al. 2022).

Dexamethasone (DEX), a synthetic GC, has been employed to investigate the cellular and molecular mechanisms of MA and assess the catabolic effects of GCs (Liu et al. 2016). The extent of MA improvement has been explored using a skeletal muscle-wasting model with a high dose of DEX on skeletal myotubes (Qin et al. 2013). Recently, researchers have begun exploring the potential effects of medicinal plant extracts in MA (Bagherniya et al. 2022).

One such plant is *Luffa cylindrica* Roemer (LCR), commonly known as sponge gourd, which belongs to the Cucurbitaceae family. LCR has been traditionally used in Korean medicine for various therapeutic effects, including fever reduction and promoting hemostasis. It contains functional components such as phenolic acid, flavonoids, anthocyanins and ascorbic acid, saponin, and vitamin A. Previous studies have reported that among phenolic acids, gallic acid and uritin B are effective for muscle atrophy. Gallic acid has been reported to increase muscle differentiation ability(Hong et al. 2020), uritin B was reported to suppress muscle atrophy by suppressing muscle atrophy factors, inducing protein synthesis, and suppressing protein degradation (Rodriguez et al. 2017). Among flavonoids, epicatechin promotes muscle growth and differentiation factors and inhibits muscle atrophy caused by aging (Gutierrez-Salmean et al. 2014). Quercetin inhibited muscle atrophy factors and suppressed gastrocnemius muscle weakness in the DEX-induced muscle atrophy model (Chen et al. 2020). Among the group of anthocyanins known as natural pigments of the flavonoid group, delphinidin has been reported to have an anti-atrophy effect by suppressing muscle atrophy factors and the loss of gastrocnemius muscle (Murata et al. 2016). Saponin is distributed in nature and is mainly known as non-volatile, surface-active compounds in plants. Saponin extracted from the roots of *Achyranthes bidentata Blume* promotes differentiation of C2C12 cells and has been reported to have an anti-atrophy effect by increasing not only muscle diameter but also the ratio of slow muscle fibers (Shi et al. 2023). Ascorbic acid, known as Vitamin C, is an essential factor not only in animals but also in plants, and mainly functions as a redox buffer. It was reported that when ascorbic acid was supplemented in ascorbic acid-deficient mice, increased muscle atrophy factors were reduced and muscle mass and physical performance were restored (Takisawa et al. 2019). LCR extract has also demonstrated anti-inflammatory, antioxidant, and immunomodulatory activities (Pan et al. 2009; Alge et al. 2006; Zhang et al. 2020; Khajuria et al. 2007; Kao et al. 2012; Dubey et al. 2015). While the effects of LCR have been investigated in various disease models, limited research has been conducted on its impact on MA.

Studies on MA commonly employ immortalized cell lines such as C2C12 and L6, due to their convenient culturing process and rapid proliferation rates. These well-established cell lines are extensively used in research. In contrast, primary cell lines, isolated from tissues, have a limited number of passages but exhibit properties similar to the cells in vivo and retain important tissue-specific characteristics and functions (Smith and Merrick 2010).

In this study, we used an in vitro model of primary skeletal muscle treated with DEX to simulate physiological characteristics, including intracellular calcium homeostasis and muscle regenerative capacity. Our investigation focused on assessing the anti-MA effect of LCR by examining changes in cell viability, MA markers, and the diameter and number of myotubes. This study offers insights into the fundamental pathophysiological mechanisms of MA and explores the potential therapeutic effects of a natural compound with minimal side effects, even after prolonged usage. Our findings aim to identify a new drug candidate and promote the development of healthier dietary options.

## Materials and methods

### Preparation of LCR extracts

The dried fruit of LCR used in this study was purchased from Green M. P. Pharmaceutical Co. Ltd. (Gyeonggi-do, Korea) by washing, drying, and cutting into small pieces. To extract LCR, it was subjected to heat treatment with LCR 15 g and distilled water 150 ml in heating mantle and reflux cooler (Misung Scientific Co. Ltd., Gyeonggido, Korea) for 3 h at 105 °C without replenishing water. The resulting mixture was then filtered through filter papers using a pump (GAST, Benton harbor mi, Michigan, USA). After filtration, the filtrate was rapidly frozen to -70 °C and subsequently lyophilized using a freeze dryer (Ilshin BioBase Co., Ltd, Gyeonggido, Korea). The lyophilized extract was kept at -20 °C until further use.

### Isolation and culture of primary skeletal myoblasts

**Scheme 1:**
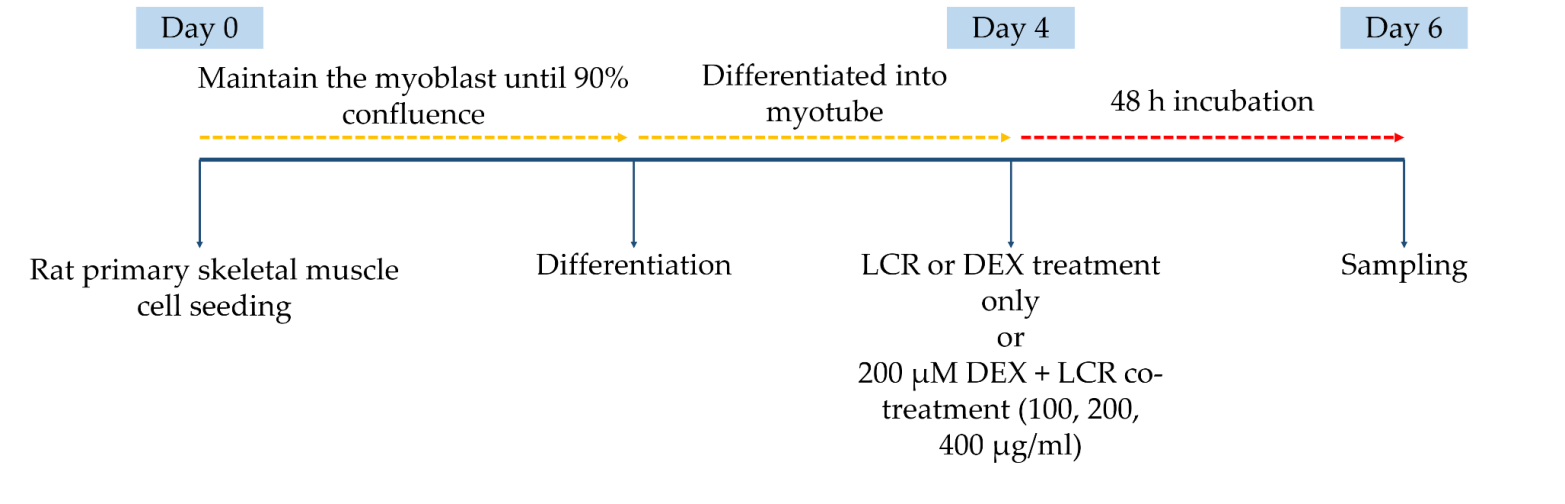
Experimental timeline of *Luffa cylindrica* Roemer (LCR) and dexamethasone (DEX) treatment

Ethical approval for this study was obtained from the Jaseng Animal Care and Use Committee (Approval number: JSR-2022-07-001-A). Primary skeletal myoblasts were obtained from 1-d-old Sprague–Dawley rats (Samtako Bio, Korea), and isolated as previously described (Musaro and Carosio 2017; Boscolo Sesillo et al. 2020). Briefly, after postnatal rats were sacrificed, the tibialis anterior muscle from the hindlimb was immediately placed in a petri dish containing cold Dulbecco’s Modified Eagle Medium (DMEM; Gibco BRL, Grand Island, NY, USA). The muscle tissue was digested using a skeletal muscle dissociation kit (Miltenyi, Bergisch Gladbach, Germany), and 10 ml of DMEM was added. The resulting suspension was then passed through a 70-μm strainer and centrifuged at 200 ×*g* for 20 min at room temperature (RT). The cell pellets were resuspended to a mixture of DMEM and Ham’s F-10 nutrient mix (Gibco BRL) at a 1:1 ratio, supplemented with 20% fetal bovine serum (Gibco BRL), 1% penicillin-streptomycin (PS, Gibco BRL), and 10 ng/ml fibroblast growth factor-basic (bFGF; Peprotech, NJ, USA). The obtained primary myoblast were seeded in culture dishes or plates coated with Matrigel (Corning, New York City, NY, USA) (Scheme 1).

### Extract treatment

Immediately before treating the prepared cells with LCR powder, a stock solution was prepared at a concentration of 100 μg/ml using 1X PBS and then diluted in culture medium to a final concentration of 100, 200, or 400 μg/ml. When cell density reached 90% confluency after myoblast seeding, the growth medium was replaced with a differentiation medium consisting of DMEM supplemented with 5% horse serum (Gibco BRL) and 1% PS. After 4 d of incubation in the differentiation medium, primary skeletal myotubes were treated with different concentrations of LCR alone or LCR and 200 μM DEX (Sigma-Aldrich, St. Louis, MO, USA), or DEX alone for 48 h (Scheme 1).

### Cell viability assay

The CCK-8 assay (CCK-8; Dojindo, Kumamoto, Japan) was used to confirm the effect of LCR on cell viability following DEX treated injury. First, primary myoblasts were seeded onto 96-well plates at 1.5 × 104 cells/100 μL. After differentiated to myotube, myotubes were treated with different concentrations of LCR with or without DEX. After incubation for 24 h, 10 μL of the CCK-8 solution was added to each well for 4 h. The absorbance of myotube was measured at 450 nm using a microplate reader (Epoch, BioTek, Winooski, VT, USA). Cell viability was calculated as the percentage of surviving neurons relative to the blank with following calculation formulas; Cell viability % = OD (sample) / Mean OD (blank) × 100.

### Western blotting

Following the treatment, the myotubes were homogenized in RIPA buffer supplemented with phosphatase and protease inhibitors (Millipore, Burlington, MA, USA) for 30 min. Protein lysates were then separated using an 8% SDS-PAGE (sodium dodecyl sulfate–polyacrylamide gel) and then transferred onto a polyvinylidene difluoride membrane (Millipore) at 100 V for 90 min. The membrane was blocked in 5% nonfat skim milk (BD Biosciences, Franklin Lakes, NJ, USA) for 1 h at RT. Subsequently, the membrane was incubated with primary antibodies (Table 1) and then with secondary antibodies for 2 h at RT. Protein bands were visualized on an Amersham Imager 600 (GE Healthcare Life Sciences, Uppsala, Sweden) imaging system using an enhanced chemiluminescence (ECL) system (Bio-Rad, Hercules, CA, USA). Quantification of protein levels was performed using ImageJ (NIH, Bethesda, Maryland, USA).

**Table 1:** Primary antibodies used for western blot and immunocytochemistry

Antibody	Company	Product no.	Dilution
Myosin Heavy Chain (MHC)	R &D Systems (Minneapolis, Minnesota, USA)	MAB4470	1:500
Atrogin-1	Abcam (Cambridge, Cambridgeshire, U.K)	Ab168372	1:1000
MURF1	Invitrogen (Waltham, Massachusetts, USA)	PA5-76695	1:1000

### Immunocytochemistry & myotue diameter measurement

Following stimulation, the myotubes were fixed using 4% paraformaldehyde for 30 min at RT and permeabilized with 0.2% Triton X-100 in phosphate-buffered saline for 10 min. The myotubes were blocked with 2% normal goat serum for 1 h before incubation with primary antibodies (Table 1) and then treated with fluorescein isothiocyanate (FITC)-conjugated secondary antibodies (Jackson; west grove, Pennsylvania, USA) for 2 h at RT. Finally, the nuclei were stained with 4′, 6-diamidino-2-phenylindole (DAPI) for 10 min at RT. The stained cells were visualized using a confocal microscope at 100× or 400× magnification (Nikon, Tokyo, Japan). Myotube diameter was measured with reference to previous studies (Rommel et al. 2001). Imaging was performed randomly under blind conditions in 10 fields for each group, 10 myotubes were measured per field. Myotube diameters were measured using Image J software (NIH, Frederick, MD, USA).

### Real-time polymerase chain reaction (PCR)

After DEX stimulation, TRIzol (15,596,018, Ambion) was added to each well for RNA extraction. The extracted RNA was reverse transcribed to cDNA using an Accupower RT PreMix (Bioneer, Daejeon, Korea). Real-time PCR was performed using SYBR Green Master Mix (170-8882AP; Bio-Rad). The primer sequences used for PCR are listed in Table 2.

**Table 2:** List of primer sequences used for Real-time PCR

Primer	Forward (5’-3’)	Reverse
*Atrogin-1*	GTCTCACGATCACCGACCTG	ATCTGCCGCTCTGAGAAGTG
*MURF1*	GGAGAAGCTGGACTTCATCG	CTTGGCACTCAAGAGGAAGG
*GAPDH*	CCCCCAATGTATCCGTTGTG	TAGCCCAGGATGCCCTTTAGT

### Statistical analysis

The results are expressed as the mean ± SD. Statistical analyses among groups were performed by one-way analysis of variance (ANOVA) followed by a Tukey’s test using GraphPad Prism software (California, CA, USA). Differences were considered statistically significant at the following p-values: #p < 0.05, ##p < 0.01, ###p < 0.001 vs. blank group, *p < 0.05, **p < 0.01, ***p < 0.001 vs. DEX group.

## Results

### LCR enhances cell viability and protects against DEX-induced muscle atrophy in primary myotubes

**Fig. 1:**
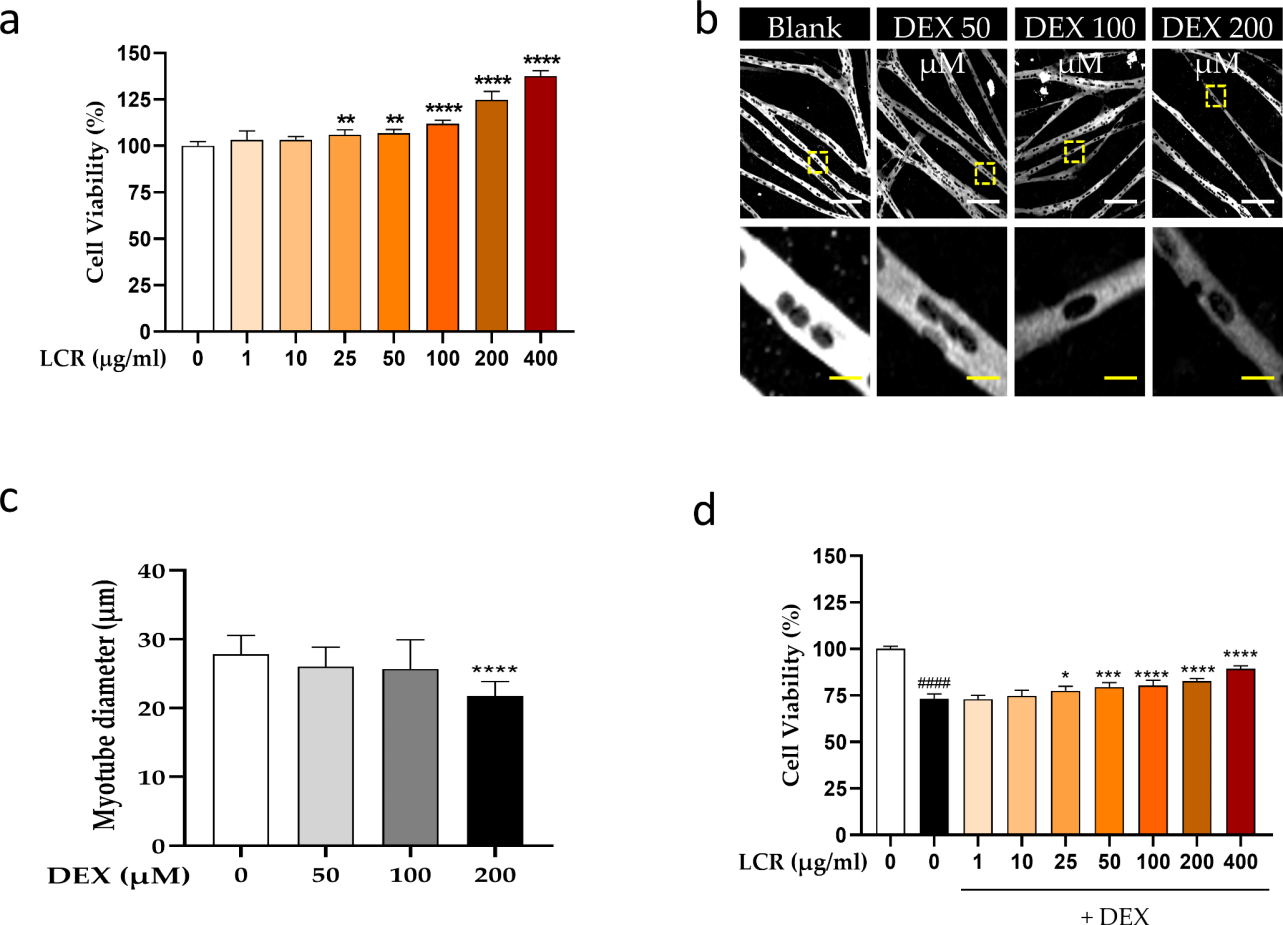
Potential protective effect of ***Luffa cylindrica*** Roemer (LCR) by increasing cell viability in primary myotubes (**a**) Cell viability of primary myotubes treated with different concentrations of LCR for 48 h (n = 6). (**b**) Representative images of myotubes treated with 50, 100, or 200 μM DEX to set up an in vitro model of MA. White scale bar, 200 μm, yellow scale bar, 20 μm. (**c**) Quantitative analysis of myotube number and diameter following treatment with 50, 100, or 200 μM DEX (n = 10). (**d**) Cell viability of primary myotubes treated with DEX treatment only or DEX and different concentrations of LCR for 48 h (n = 6). Data are expressed as means ± SD. The results were evaluated using a one-way analysis of variance (*p < 0.05, **p < 0.01, ***p < 0.001, and ****p < 0.0001 vs. DEX group; ####p < 0.0001 vs. blank group)

Initially, primary myoblasts were prepared from the tibialis anterior (TA) muscle of 1-d-old rats and their differentiation into myotubes prompted. When initiating fusion to form the myotubes, spontaneous beating occurred, forming myotube-like structure (Video S1). To determine whether LCR possesses cytotoxicity towards differentiated myotubes, we performed a CCK-8 assay. Following treatment with LCR at varying concentrations (1 to 400 μg/ml), cell viability increased significantly for all concentrations > 25 μg/ml (Fig. 1a). First, we conducted ICC experiment to confirm the effect of LCR under conditions where atrophy occurs due to DEX and reduces cell viability rate. Primary myotubes were treated with 50, 100, and 200 μM DEX to establish optimal concentrations of DEX, referring to previous references (Wang et al. 2021) (Fig. 1b). Subsequently, we evaluated myotube diameter as an indicator of MA induction. The results revealed that there was no significant difference at 50, 100 μM, but only myotube diameter treated with 200 μM DEX was significantly reduced (Fig. 1c). Therefore, we used DEX 200 μM-treated primary myotubes as an in vitro model of MA. When myotubes were subjected to DEX treatment alone, cell viability was significantly decreased, while LCR co-treatment restored cell viability in a dose-dependent manner in the model (Fig. 1d). Our findings suggest that LCR is not cytotoxic to primary myotubes and has potential protective activity against DEX-induced MA in myotubes.

### LCR promotes myotube formation in the primary skeletal muscle of rats

**Fig. 2:**
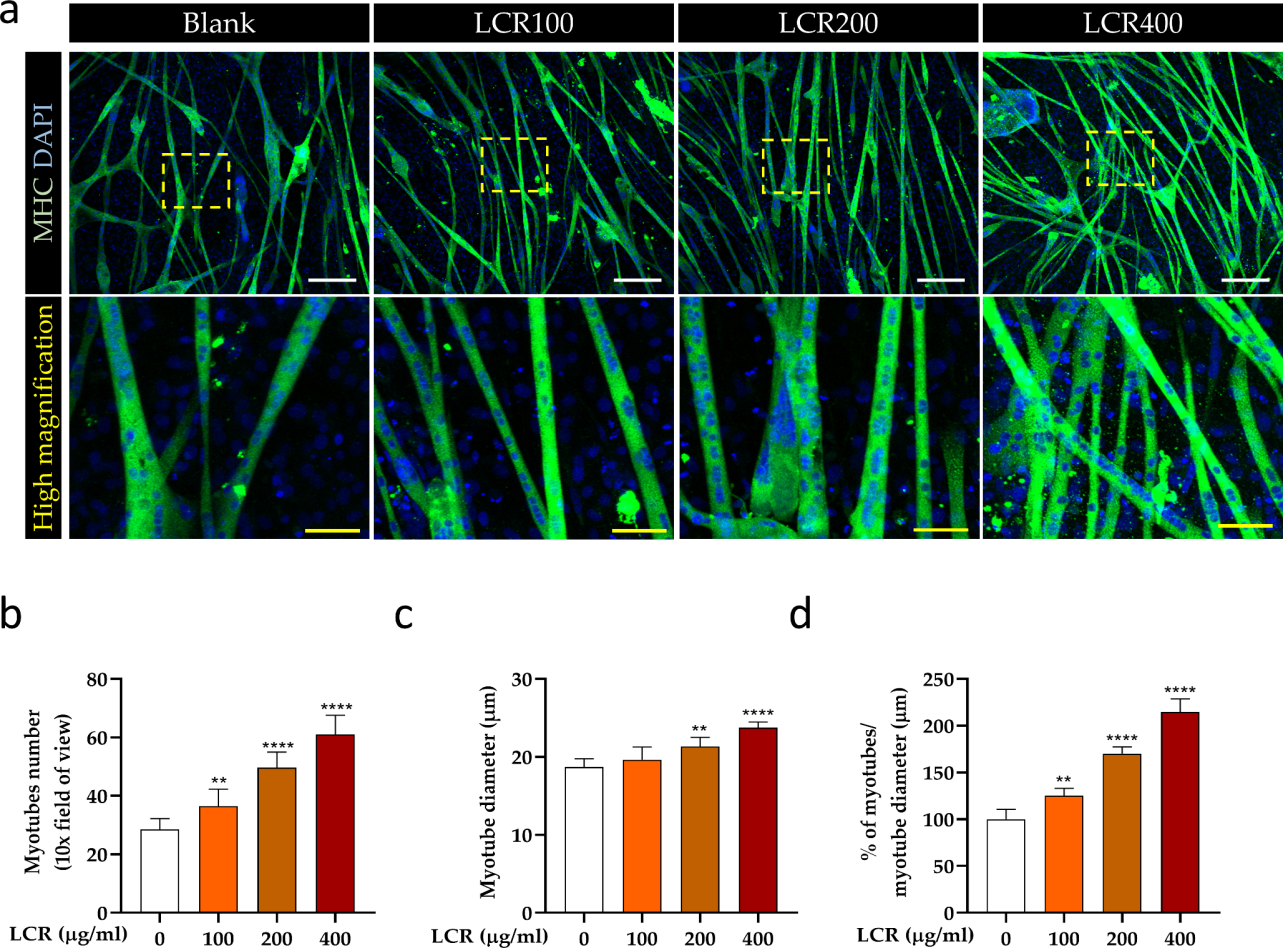
Facilitating effect of LCR on myotube formation in rat primary skeletal muscles (**a**) Immunofluorescence images showing MHC (green) expression and DAPI (blue). White scale bar, 200 μm, yellow scale bar, 50 μm. (**b**, **c**) Quantitative analysis of myotube number and diameter following LCR exposure (n = 10). (**d**) The percentage of the number of myotubes to their diameter (n = 10). Data are expressed as means ± SD. The results were evaluated using a one-way analysis of variance (**p < 0.01, ***p < 0.001, and ****p < 0.0001 vs. blank group)

To investigate the effect of LCR in promoting myotube formation, staining for MHC was performed after exposure to LCR for 48 h without DEX treatment. LCR treatment resulted in the formation of more MHC + myotubes and thicker myotubes in a dose-dependent manner (Fig. 2a). Further quantitative analysis substantiated these findings, as LCR treatment demonstrated a significant and progressive improvement in myotube formation, with an increased number observed at higher concentrations of LCR (Fig. 2b). Moreover, the diameter of the myotubes increased in a dose-dependent manner, with a significant enhancement evident upon treatment with 200 and 400 μg/ml LCR (Fig. 2c). When quantitatively analyzed by calculating the percentage of the myotubes with the average myotube diameter according to LCR concentration, a significant concentration-dependent increase was observed in the LCR-treated groups (Fig. 2d). Collectively, these observations suggest that LCR facilitates myotube formation by enhancing both the number and diameter of myotubes.

### LCR suppresses DEX-induced upregulation of Atrogin-1 and MuRF1 in primary myotubes

**Fig. 3:**
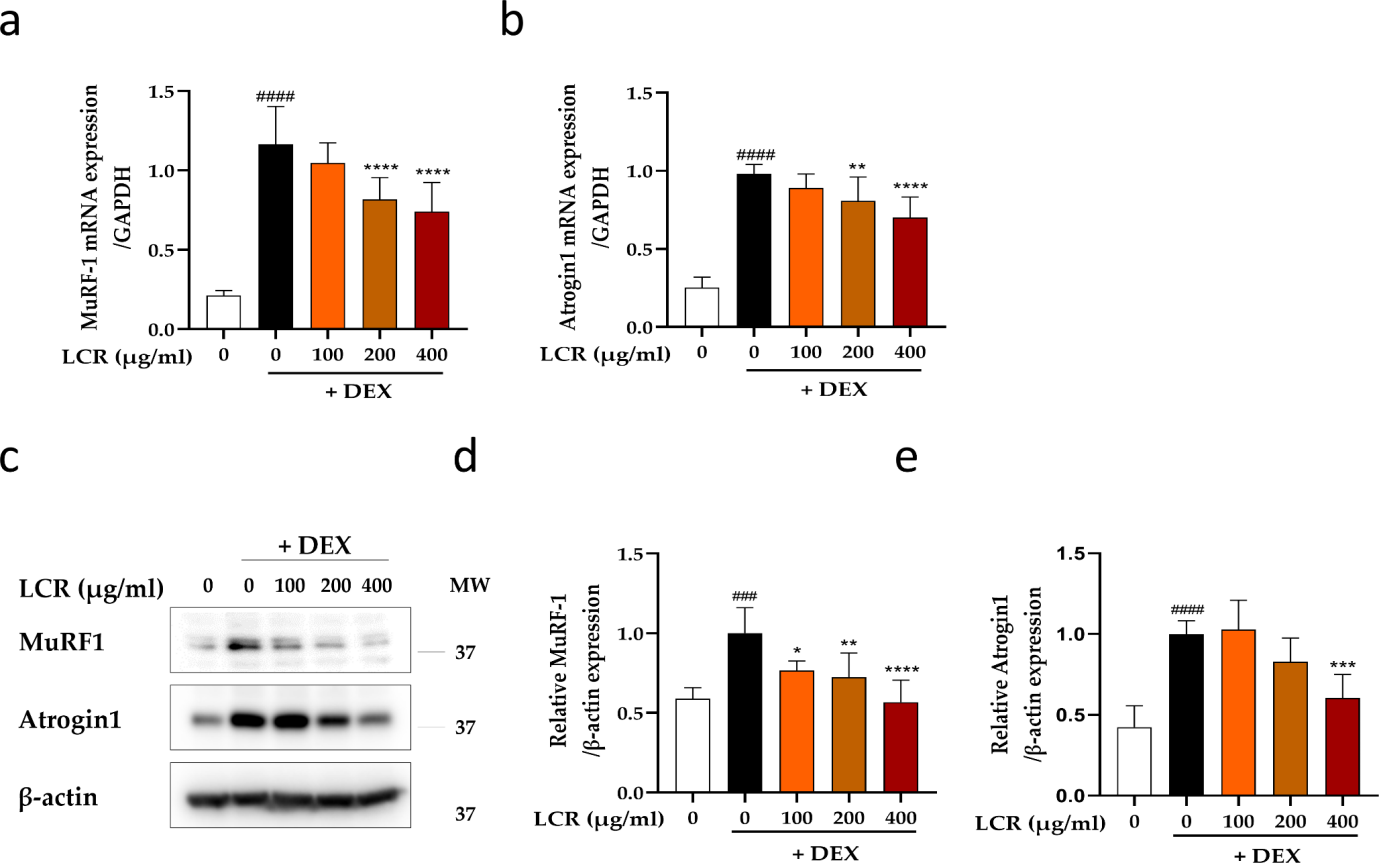
Inhibitory effect of LCR on DEX-induced upregulation of Atrogin-1 and MuRF1 in primary myotubes (**a**, **b**) mRNA expression levels of *MuRF1* and *Atrogin-1* analyzed by real-time PCR. (**c**) Representative western blot bands for MuRF1 and Atrogin-1 in each group. (**d**, **e**) Relative quantification of the MuRF1 and Atrogin-1 protein in each group (n = 5). Data are expressed as means ± SD. The results were evaluated using a one-way analysis of variance (*p < 0.05, **p < 0.01, ***p < 0.001, and ****p < 0.0001 vs. DEX group; ###p < 0.001, and ####p < 0.0001 vs. blank)

To evaluate the inhibitory effect of LCR on MA-related ubiquitin ligases, namely MuRF1 and Atrogin-1 (Foletta et al. 2011), we examined their mRNA and protein levels in primary myotubes. As shown in Fig. 3a and b, the expression levels of *MuRF1* and *Atrogin-1* mRNA were significantly increased following DEX treatment compared with those in the blank group, whereas LCR decreased these expression levels dose dependently, especially at 200 and 400 μg/mL concentrations. To confirm these findings at the protein level, further analysis through western blotting was performed (Fig. 3c) and the protein expression levels of MuRF1 and Atrogin-1 were significantly increased by DEX treatment compared with those in the blank group, whereas LCR decreased these levels concentration dependently (Fig. 3d, e). Notably, Atrogin-1 protein expression significantly decreased only at a concentration of 400 μg/ml (Fig. 3e). Our findings suggest that LCR administration restored the expression of Atrogin-1 and MuRF1 to normal levels, effectively counteracting the DEX-induced upregulation.

### LCR restores the number and diameter of primary myotubes reduced by DEX-induced MA

**Fig. 4:**
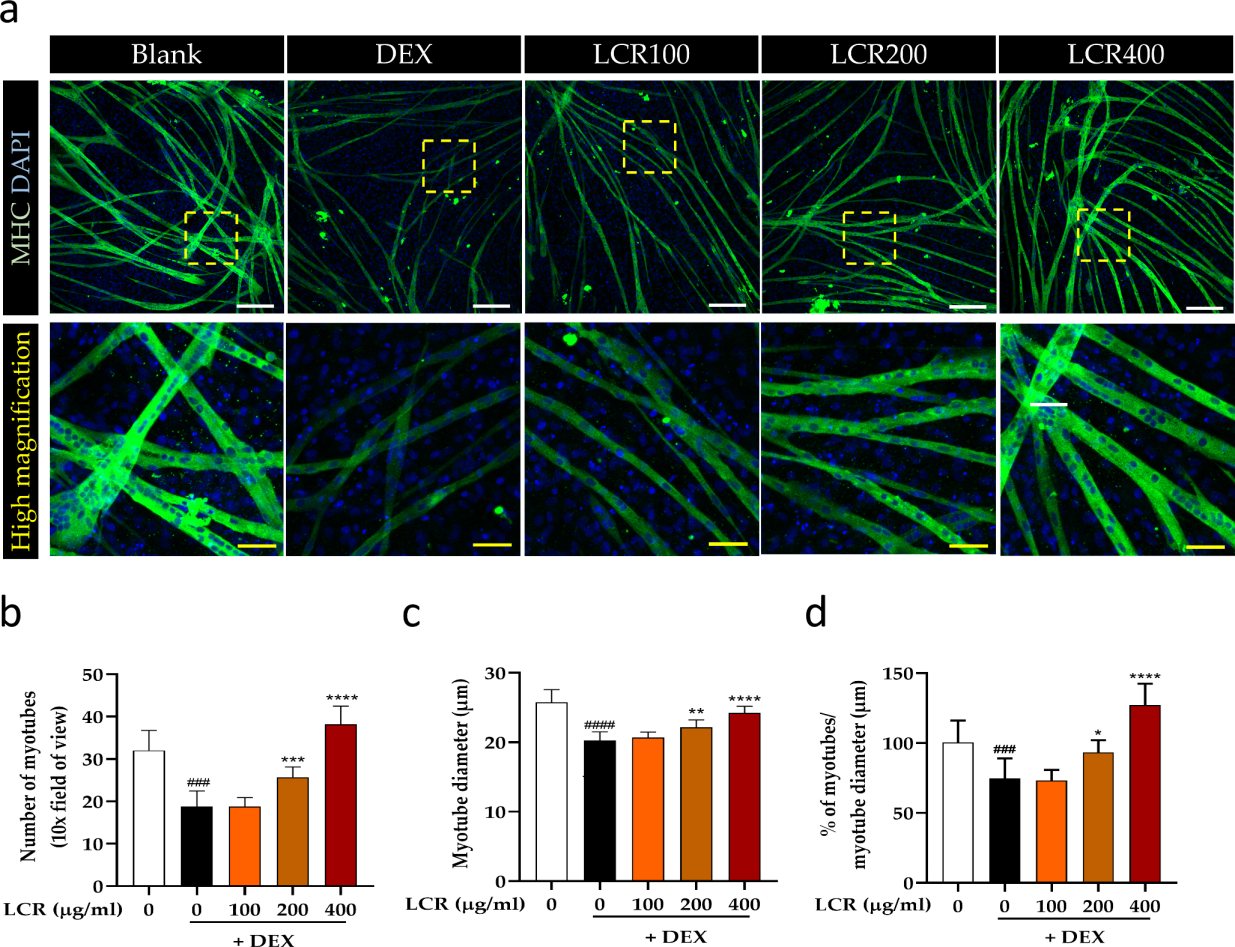
Restorative effect of LCR on DEX-induced MA in primary myotubes (**a**) Immunofluorescence images of MHC (green) expression and DAPI (blue). White scale bar, 200 μm; Yellow scale bar, 50 μm. (**b**, **c**) Quantitative analysis of myotube number and diameter in each group under LCR and DEX conditions (n = 10). (**d**) The ratio of the number of myotubes to their diameter (n = 10). Data are expressed as means ± SD. The results were evaluated using a one-way analysis of variance (*p < 0.05, **p < 0.01, ***p < 0.001, and ****p < 0.0001 vs. DEX group; ###p < 0.001, and ####p < 0.0001 vs. blank)

DEX treatment resulted in the induction of MA by upregulating the expression of Atrogin-1 and MuRF1, consequently leading to a reduction in muscle mass with decreased myotube diameter and thickness (McRae et al. 2017; Castillero et al. 2013). In light of these observations, we evaluated the potential protective effect of LCR against DEX-induced MA by confocal analysis. Primary myotubes were stained for MHC, which indicated the final stage of myogenesis (Fig. 4a). DEX treatment significantly decreased both the myotube number and diameter compared with those in the control group. However, LCR effectively restored these parameters in a dose-dependent manner (Fig. 4b, c). Furthermore, the ratio of the number of myotubes to their diameter, indicative of myotube quality, was significantly improved at LCR concentrations of 200 and 400 μg/ml (Fig. 4d). These results demonstrate that LCR treatment led to the recovery of myotube number, diameter, and overall myotube quality, thereby mitigating the detrimental effects of DEX on primary myotubes.

## Discussion

In the present study, we evaluated the effects of LCR on skeletal muscle differentiation and its protective role against GC-induced MA. Treatment with LCR resulted in an increased number of myotubes containing MHC, a specific marker of mature myotubes, with a concomitant increase in their diameter. These findings demonstrate that LCR mitigates the detrimental effects of DEX on primary myotubes.

DEX can induce either MA or hypertrophy, depending on the stage of differentiation of C2C12 cells in vitro. Treatment with DEX during the myoblast stage promotes myoblast proliferation, myotube enlargement, and expression of MHC, a protein associated with terminal differentiation while reducing the expression of atrophy markers, such as myostatin and Atrogin-1, thereby inducing hypertrophy. However, when myotubes are exposed to DEX, the expression of atrophy markers, myostatin, and Atrogin-1, increases in a concentration-dependent manner, whereas the expression of myoblast markers, pax7, and MHC, decreases. This suggests that the process of muscle atrophy is influenced by the ubiquitin-proteasomal pathway (Guerriero and Florini 1980; Han et al. 2017). Local injection of DEX alone does not induce muscle atrophy, as confirmed by histological evaluations, reduction in local inflammatory cytokines, and increased contractile tension, indicating beneficial effects on muscle strain (Hakim et al. 2005). These findings indicate that the use of DEX in muscle-related studies should be carefully considered based on the specific objectives. The results of our study revealed that LCR, in primary myotubes, reduces the expression of Atrogin-1 and MuRF1 by inhibiting the ubiquitin-proteasomal pathway activated by DEX. Additionally, LCR increases the number and diameter of myotubes, suggesting it as a natural compound with promising potential for protecting skeletal muscle.

From previous studies, LCR is known to contain a flavonoid called quercetin. Quercetin is not known to cause muscle atrophy induced by dexamethasone, but its anti-atrophy effect was confirmed by reducing the Atrogin1 and MuRF1 in obese mice treated by a high fat diet, and quercetin glycoside was known to increase the ratio of the gastrocnemius muscle to boby weight in mice treated with dexamethasone. Therefor, this study may be assumed that the protective effect of LCR against dexamethasone induced muscle atrophy is due to the anti-atrophy effect of quercetin (Dubey et al. 2015; Otsuka et al. 2019; Davis et al. 2009).

This study has some limitations. Although we assessed MHC levels, which are expressed in myotubes and myofibers and are most abundant in myocytes, we did not analyze other muscle differentiation markers, such as MyoD and myogenin. However, considering the number and diameter of MHC-stained myotubes at the final stage of muscle differentiation, we hypothesize that LCR promotes skeletal muscle differentiation in a concentration-dependent manner. However, further studies are needed for the investigation of the molecular mechanism of skeletal muscle development by LCR.

In conclusion, our study provides evidence that LCR promotes skeletal muscle differentiation and exerts an anti-muscle atrophy effect. Furthermore, this study highlights the antioxidant, anti-inflammatory, and immunomodulatory properties of LCR and evaluates its potential for protecting against DEX-induced MA. These results highlight the potential therapeutic value of LCR in counteracting muscle atrophy and preserving skeletal muscle integrity.

### Electronic supplementary material

Below is the link to the electronic supplementary material.

**Supplementary Material 1: Video S1**: Live-cell image of primary rat skeletal myotube

## Data Availability

The datasets generated during and/or analysed during the current study are available from the corresponding author on reasonable request.
